# Calcium Electrochemotherapy and Challenges in Combined Treatment with Dendritic Cell Vaccination

**DOI:** 10.3390/pharmaceutics17070804

**Published:** 2025-06-21

**Authors:** Eivina Radzevičiūtė-Valčiukė, Austėja Balevičiūtė, Augustinas Želvys, Karolina Suveizdė, Auksė Zinkevičienė, Vytautas Kašėta, Veronika Malyško-Ptašinskė, Neringa Dobrovolskienė, Vita Pašukonienė, Jurij Novickij, Irutė Girkontaitė, Vitalij Novickij

**Affiliations:** 1 Department of Immunology and Bioelectrochemistry, State Research Institute Centre for Innovative Medicine, LT-08410 Vilnius, Lithuania; austeja.baleviciute@ki.se (A.B.); augustinas.zelvys@imcentras.lt (A.Ž.); aukse.zinkeviciene@googlemail.com (A.Z.); veronika.malysko-ptasinske@vilniustech.lt (V.M.-P.); irute.girkontaite@imcentras.lt (I.G.); 2 Faculty of Electronics, Vilnius Gediminas Technical University, LT-10105 Vilnius, Lithuania; jurij.novickij@vilniustech.lt; 3 Laboratory of Immunology, National Cancer Institute, LT-08660 Vilnius, Lithuania; karolina.zilionyte@nvi.lt (K.S.); neringa.dobrovolskiene@nvi.lt (N.D.); vita.pasukoniene@nvi.lt (V.P.); 4 Department of Stem Cell Biology, State Research Institute Centre for Innovative Medicine, LT-08410 Vilnius, Lithuania; vytautas.kaseta@imcentras.lt

**Keywords:** electroporation, dendritic cells, murine, carcinoma, myeloma, pulsed electric fields

## Abstract

**Background/Objectives:** Electrochemotherapy (ECT) is a reliable and potent technique for managing primary tumors; however, significant efforts are being made to characterize and improve the systemic immune response, which is crucial for metastasis prevention. Current evidence suggests that the advancement of ECT will depend on its integration with complementary immunomodulatory methods. **Methods:** In this study, we examined the combined effects of calcium-based electrochemotherapy (CaECT, 1.3 kV/cm × 100 µs, eight pulses delivered at 1 Hz repetition frequency) with dendritic cell vaccination (DCV). Lewis lung carcinoma (LLC1) was used as a tumor model. We characterized the effects of CaECT alone and in combination with DCV therapy on tumor growth, analyzed the changes in immune cell subpopulations, and studied the humoral immune response dynamics on day 10, 20, and 30. Given the limited effect of DCV, additional experiments were conducted with the chemotherapeutic drug cyclophosphamide (CP), known for its immunomodulatory properties. **Results:** Although CaECT demonstrated potent antitumor activity and induced a significant immune response, its combination with DCV did not result in enhanced therapeutic efficacy. The combination of CP also failed to improve median survival. **Conclusions:** It is concluded that CaECT is a promising alternative to standard ECT involving bleomycin or cisplatin. However, further optimization is necessary to enhance the therapeutic synergy of CaECT when combined with DCV.

## 1. Introduction

A pulsed electric field (PEF) used in electroporation (EP) induces changes in the transmembrane potential, resulting in the formation of hydrophilic pores and thereby increasing the permeability of the cell membrane through a physical, non-thermal mechanism. The induced cell permeability facilitates intracellular delivery of initially impermeable or low-permeability molecules, including dyes, ions, drugs, and macromolecules, e.g., proteins and nucleic acids (RNA, DNA) [1,2,3,4,5]. The impact on the cell membrane can be regulated by altering the characteristics of the electric pulses, such as their intensity, repetition rate, duration, and quantity. The cell plasma membrane permeabilization can be either reversible (RE) or irreversible (IRE) when higher intensities of PEF are used, causing permanent membrane disruption and subsequent cell death [6]. The RE permeabilization is being employed for molecular delivery of poorly permeable molecules (e.g., bleomycin) for treatment of cancer—a technique known as electrochemotherapy (ECT). This approach enhances drug cytotoxicity while allowing substantial reduction of the required drug dose (several orders of magnitude), compared to conventional chemotherapy [7,8]. ECT is typically administered using microsecond pulse protocols (100 µs × 8) and has been proven to be a reliable and efficacious cancer therapy across in vitro studies, animal models, and clinical applications [6,9,10,11].

As a standard practice, bleomycin and cisplatin are frequently used in electrochemotherapy procedures as cytotoxic agents [12,13,14]. More recently, calcium electrochemotherapy (CaECT) or calcium electroporation (CaEP) (terms used interchangeably in the literature) has emerged as a promising alternative. This approach is gaining increasing attention due to its safety, efficacy, and potential to replace conventional chemotherapeutic agents in ECT [15]. Calcium-based ECT offers several advantages over traditional approaches. Unlike chemotherapeutic drugs, calcium does not induce resistance in cancer cells (no such reports to date) and is a safe, inexpensive, and widely available compound [16,17]. The therapeutic efficacy of Ca-based treatments is primarily attributed to the ion’s biophysical and regulatory roles in cellular function. Calcium ions serve as essential second messengers, playing a central role in critical cellular functions including gene transcription, proliferation, metabolism, and programmed cell death [15,18,19]. To maintain viability, cells strictly regulate intracellular calcium levels. However, CaECT-induced cell membrane permeability leads to elevated intracellular calcium concentration, causing ATP depletion [20,21], mitochondrial dysfunction, and ultimately cell death [15,19,22,23,24].

Recent studies have shown that CaECT induces a systemic immune response. An in vitro study revealed that CaECT enhances the release of damage-associated molecular pattern (DAMP) molecules, leading to immunogenic cell death [25,26,27]. Concurrently, in vivo calcium-based ECT studies showed that the immune system activation is crucial for effective treatment [26]. Positive changes in the anti-tumor immune response were also reported in an in vivo calcium IRE study [28], resulting in increased T cell numbers, fewer myeloid-derived suppressor cells (MDSCs), and elevated levels of tumor-specific antibodies.

Clinical research has also demonstrated that CaECT can trigger a systemic immune response, leading to both local tumor control and distant tumor regression in the patient with malignant melanoma [29,30,31]. CaECT alone prolonged remission in approximately 50% of patients diagnosed with highly aggressive mucosal head and neck squamous cell carcinoma. Furthermore, CaECT had been shown to be easily adaptable to clinical requirements, enabling rapid implementation and patient discharge shortly after treatment [32].

However, the formation of metastases beyond the treated area remains the major therapeutic challenge in post-electroporation therapy. To enhance the systemic effects of EP-based treatment, combination strategies incorporating immunotherapeutic agents are being explored with the aim of achieving synergistic outcomes. Several studies have conducted the integration of EP with immune checkpoint blockade antibodies targeting programmed cell death protein 1 (anti-PD1) [33,34], cytotoxic T-lymphocyte-associated protein 4 (anti-CTLA4) [35,36,37], as well as the electrotransfer of plasmids encoding pro-inflammatory cytokines interleukin-2 and interleukin-12 (IL-2 and IL-12) [21,26,38,39,40], granulocyte-monocyte colony-stimulating factor (GM-CSF) [41], and stimulator of interferon gene (STING) agonists [42], demonstrating encouraging results with systemic responses. In parallel, dendritic cell (DC)-based vaccines (DCVs) have emerged over the past decade as an immunotherapeutic approach in cancer treatment [43,44].

DCs represent a rare but critical subpopulation of leukocytes that function as antigen-presenting cells (APCs). Operating within peripheral tissues, DCs capture, process, and transport antigens to secondary lymphoid organs, where they present antigen-derived peptides that are sourced from pathogens or the host, involving tumor antigens to antigen-specific naïve T lymphocytes via major histocompatibility complex (MHC) molecules [45,46,47,48]. As APCs, DCs play a crucial role in the stimulation, regulation, and maintenance of various immunological responses and, most importantly, the promotion of antitumor immunity through interactions with other immune cells [45,49]. Due to their central role in immune surveillance, DCs are key candidates for cancer immunotherapy, particularly in DCVs, where patient-derived DCs are loaded ex vivo with tumor-associated antigens (TAAs) and reinfused to trigger a tumor-specific immune response [50].

Currently, numerous preclinical and clinical studies [51] have provided evidence supporting the efficacy of DCVs, both as monotherapies and in combination with various immunostimulatory agents, including synthetic peptides, exosomes, Toll-like receptor agonists, and other modulatory molecules [50,51,52,53]. Most DCVs are autologous and monocyte-derived, although recent work has explored the use of alternative DC subtypes. Typically, tumor antigens are presented to patient-derived DCs outside the body before they are reintroduced. A growing trend in DCV development is the personalization of vaccines using tumor-specific neoantigens to enhance specificity and efficacy [51]. Numerous in vivo studies have demonstrated the therapeutic potential of dendritic cell vaccines. For instance, Nimanong et al. combined DC vaccines with a costimulatory cocktail, achieving remission of large murine tumors [54], while Moreno Ayala et al. enhanced antitumor responses by combining DCVs with FOXP3-targeting peptide [55] and TLR9 agonists [56]. Liu et al. tested DC vaccines with glioma-derived exosomes and iNKT cell agonists, inducing antitumor immunity in rats [57], and Escriba-Garcia et al. combined DC vaccines with α-GalCer, achieving 100% tumor-free survival in mice with B-cell lymphoma [58]. Additional synergistic effects were reported when DCVs were combined with lenalidomide [59], a CSF1R inhibitor [60], CCL21-overexpressing DCs [61], and chitosan nanoparticles targeting LGALS1 [62]. Collectively, these studies underscore the therapeutic promise of DCVs in cancer treatment [50]; however, there are no immunology-focused in vivo articles covering both dendritic cell vaccines and CaECT. Thus, the synergistic potential of DCV therapy combined with pulsed electric field-based treatment modalities remains largely uninvestigated yet.

At the same time, cyclophosphamide (CP) is an alkylating agent; it is extensively utilized in standard cancer treatments alongside other drugs for the treatment of breast cancer, malignant lymphomas, multiple myeloma, and neuroblastoma [63,64]. Its mechanism of action involves cytotoxic effects through DNA strand cross-linking, which inhibits cell division and promotes apoptosis in rapidly proliferating cells [65,66]. It is also reported that CP plays a crucial role in immunomodulation, when administered in low doses, as it can deplete regulatory T cells (Tregs), thereby enhancing naturally primed T cells to boost antitumor immunity, as demonstrated in both human [67,68,69,70] and mouse studies [71]. A recent murine neuroblastoma study confirmed that a low-dose CP treatment resulted in the depletion of Tregs specifically within the tumor microenvironment, along with phenotypic alterations in other T cell subsets [72]. The immunomodulatory effects of CP highlight its potential to enhance cancer treatment by boosting antitumor immunity, with different therapies in particular. This synergy can improve efficacy by promoting a robust and targeted immune response [67,73,74].

This study aimed to evaluate whether combination of the DCV with standard microsecond-range CaECT (1.3 kV/cm, 100 μs, eight pulses at 1 Hz) yields synergistic effects, enhancing treatment efficacy, prolonging overall survival, and suppressing tumor growth in the LLC1 tumor model. We assessed the modulation of the antitumor immune response induced by CaECT alone and in combination with the DCV. Additionally, CP was used in conjunction with the DCV and CaECT to explore their combined effects on tumor growth suppression and overall survival.

## 2. Materials and Methods

### 2.1. Mice and Tumor Induction

Female C57BL/6 mice were bred and carefully maintained under controlled conditions at the mouse facility of the State Research Institute Centre for Innovative Medicine, located in Vilnius, Lithuania. The animals were housed in standard cages with regulated temperature, humidity, and a 12 h light/dark cycle, ensuring their well-being throughout this study. For this experiment, tumors were induced in healthy six- to eight-week-old mice by administering a subcutaneous (s.c.) injection of 1 × 10^6^ Lewis lung carcinoma cells. When tumors reached 100 mm^3^, treatment was applied (Day 0).

The Lithuanian State Food and Veterinary Service granted approval for all experimental protocols (Approval no. G2-145). Throughout this study, strict compliance with the Guide for the Care and Use of Laboratory Animals was maintained to ensure ethical treatment and welfare of the animals.

### 2.2. Electroporation Procedure

In this experiment, a square-wave pulse generator, developed at VILNIUS TECH (Vilnius, Lithuania), was utilized to deliver the electric pulses. The pulses were applied through adjustable gap parallel plate stainless steel electrodes, with a 3 mm gap employed during the procedures. Mice received treatment with pulsed electric fields set at 1.3 kV/cm, delivered as eight pulses each lasting 100 μs, at a frequency of 1 Hz. The charging voltage was maintained at 390 V, corresponding precisely to the desired voltage-to-gap ratio of 1.3 kV/cm.

Initially, the backs of the mice were shaved and depilated using an 8% aqueous solution of sodium sulfide (Na_2_S), followed by thorough rinsing with water to ensure complete removal of hair. Prior to the treatment procedures on Day 0, the mice were anesthetized with a mixture of 3% isoflurane and oxygen gas to ensure immobilization and minimize discomfort. A single intratumoral injection of 0.25 M calcium chloride (CaCl_2_) dissolved in 0.9% sodium chloride (NaCl) solution was administered, amounting to approximately half of the tumor’s volume. Immediately following the injection, the mice were subjected to PEF treatment.

To monitor tumor progression, volumetric assessment of the growth dynamics was performed. Tumor dimensions were measured using a high-precision digital caliper at the tumor’s central axis every 48 to 72 h post-treatment. Tumor volume (V, in mm^3^) was computed using the ellipsoid formula: V = (L^2^ × W × π)/6, where L is tumor length, W is tumor width, and *π* approximated as 3.1416 [75,76].

Animals were maintained under standard laboratory conditions until either experimental endpoint were reached or tumor volume exceeded 2000 mm^3^, necessitating humane euthanasia by cervical dislocation. Mice exhibiting complete recovery after the treatment were monitored for a period of up to 60 days.

### 2.3. Dendritic Cell Vaccine Preparation

Murine dendritic cell vaccines were prepared following an optimized Lutz et al. protocol [77]. Bone marrow cells were isolated from healthy C57BL/6 (8–12 weeks old) and differentiated for 7 days using GM-CSF (12 ng/mL, Miltenyi Biotec, Bergisch Gladbach, Germany) and IL-4 (3%, IL-4 secreting cell supernatant). Dendritic cell maturation was induced by 24 h culture with LLC1 (15 μg/mL) and *Escherichia coli* lipopolysaccharide (LPS; 1 μg/mL, O55:B5 serotype, Sigma, Burlington, MA, USA), maintaining the same concentration of GM-CSF and IL-4. CD80, CD86, CD40 PD-l MHCII, and MHC expression was measured to determine DC maturation. The quality of prepared dendritic cells was assessed phenotypically.

### 2.4. Experimental Scheme

**Figure 1:**
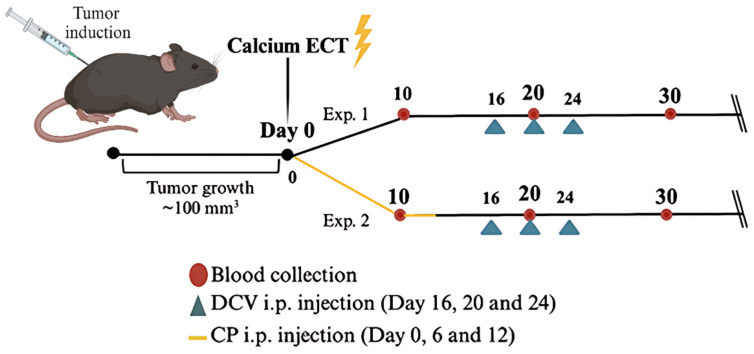
Schematic experimental design.

Two independent experiments were performed using the C57BL/6 carcinoma tumor model. In the first experiment (Exp. 1), mice were divided into three experimental cohorts: tumor-bearing controls group (CTRL), mice receiving CaECT, and mice subjected to combined treatment with CaECT followed by i.p. administration of dendritic cell vaccine (CaECT + DCV) (Figure 1, Exp. 1). In the other experiment (Exp. 2), mice were divided into five groups: tumor-bearing controls (CTRL), mice treated with cyclophosphamide, additionally combined with DCV (CP + DCV) or CaECT (CaECT + CP + DCV) (Figure 1, Exp. 2). Note that the second experiment (Exp. 2) was performed after the first one (Exp. 1) was over.

In every experiment, 10, 20, and 30 days after treatment, mouse blood was collected from the murine tail vein and allowed to clot. Serum obtained after centrifugation of the mouse blood was subsequently used to determine specific antitumor antibodies (only for Exp. 1). Mouse groups that received DCV were injected intraperitoneally (i.p.) on days 16, 20, and 24. Each mouse received 200 µL of DCV that was resuspended in RPMI 1640 growth medium without supplements. The IP injection of DCV was used as the peritoneal injection site was close to the tumor site. We did not attempt to inject DCV intratumorally because of post-electroporation necrotic scab.

In experiment 2 (Figure 1, Exp. 2), i.p. injections of cyclophosphamide were additionally used to treat mice. Each mouse received 100 µL of 2.6 mg CP that was resuspended in sterile phosphate-buffered saline (PBS) [78].

Additionally, in the C57BL/6 carcinoma tumor model study (Figure 1, Exp. 1), spleens and lymph nodes were collected from euthanized mice and used for immune cell analysis with multicolor flow cytometry.

### 2.5. Flow Cytometry

Single cells were isolated from spleens and lymph nodes using a cell strainer (70 µm). Cells were centrifuged at 400× *g* for 5 min at room temperature (RT). Afterward, tumor and lymph node cells were washed with phosphate buffer saline (PBS) and resuspended in a small amount of buffer for flow cytometry (FACS buffer; 2% fetal bovine serum (FBS) and 0.1% NaN3 in PBS). Splenocytes were treated with ammonium chloride to lyse mouse erythrocytes and were afterward also resuspended in the FACS buffer (79).

Cell surface staining was performed by incubating 1 × 10^6^ cells with a monoclonal antibodies (mAbs) master mix. Prepared samples were incubated on ice, away from light, for 30 min. Cell populations were identified by using different sets of fluorochrome-labeled mAbs and fluorescent dyes. The measurement and analysis of immune organs were performed with a BD FACS Aria III instrument (BD Biosciences, San Jose, CA, USA) and analyzed by FlowJo software 10.8.1 (BD, USA). The antibodies used in the staining and the gating strategy are presented in the Supplementary Materials (Table S1 and Figure S1). Gating and analysis strategies were applied to flow cytometry data based on a previously published article [79].

### 2.6. Determination of Antitumor Antibodies

Serum samples were collected from mice to assess the presence of antibodies targeting both surface and intracellular antigen LLC1 cells. The detection of antitumor IgG antibodies was conducted following a previously established protocol [79]. Briefly, cells were fixed with RT 2% paraformaldehyde (PFA) and permeabilized with 0.2% ice-cold Triton X-100. After permeabilization, cells were resuspended in FACS buffer and centrifuged again. The resulting cell suspensions were then passed through a 70 μm mesh filter and diluted with Fc Block reagent to prevent non-specific binding. These prepared suspensions were incubated for 1 h with mouse sera, which had been serially diluted with PBS. Thereafter, cells were washed using PBS and incubated with a goat anti-mouse IgG conjugated to AF488 (eBioscience, Invitrogen, Thermo Fisher Scientific, Waltham, MA, USA) on ice for half an hour. Cells that were maintained solely with goat anti-mouse IgG AF488 antibodies were considered for negative control. Data acquisition was carried out using the Amnis FlowSight cytometer (Amnis Luminex/MilliporeSigma, Burlington, MA, USA), and subsequent data analysis was performed with FlowJo version 10.8.1 software (BD, Ashland, OR, USA).

### 2.7. Methods of Statistical Analysis

The non-parametric Mann–Whitney–Wilcoxon test was employed to evaluate whether flow cytometry data from various organs (spleens, lymph nodes), as well as the relative concentration of antitumor antibodies, significantly differed between groups of treated and untreated mice. The log-rank (Cox-Mantel) and Gehan–Breslow–Wilcoxon tests were used to analyze mice survival data (Kaplan–Meier survival analysis), comparing groups of treated mice among each other and to the group of untreated (CTRL) mice.

A *p*-value of less than 0.05 was statistically significant (* *p* < 0.05; ** *p* < 0.005; *** *p* < 0.0005; **** *p* < 0.00005; ■—outliers). The data were analyzed using GraphPad Prism 8 software (GraphPad Software Inc., La Jolla, San Jose, CA, USA).

## 3. Results

### 3.1. Evaluation of the Phenotype of Prepared DCV

Following the DCV preparation, their quality was evaluated based on the purity and the expression of specific markers. For DCs to induce antitumor T lymphocyte response, the expression of costimulatory (CD80, CD86, CD40) and Ag-presenting (MHCII) molecules is required. The expression of these markers on DCs was assessed before and after the maturation stage to verify whether the maturation conditions led to functional activation (maturation) of dendritic cells. The expression of the PD-L1 molecule, which has immunosuppressive effects, was also assessed.

**Figure 2:**
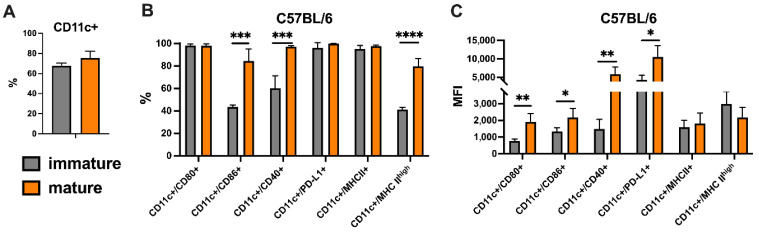
Phenotype of bone marrow-derived dendritic cells from C57BL/6 mice. (**A**) The purity of prepared DCs according to CD11c marker expression. (**B**) The percentage of CD80, CD86, CD40, PD-L1, and MHC-II expression in CD11c^+^ population. (**C**) Mean fluorescence intensity (MFI) of CD80, CD86, CD40, PD-L1, and MHC-II expression in CD11c^+^ population. A black asterisk (*) indicates statistically significant differences between groups, with significance levels denoted as follows: (* *p* < 0.05, ** *p* < 0.005, *** *p* < 0.0005, **** *p* < 0.00005).

The purity of the prepared dendritic cells (CD11c^+^) was 75.43 ± 6.99% (Figure 2A). After maturation, both the quantity of costimulatory molecules and the proportion of CD11c^+^ cells expressing them increased (Figure 2B).

Along with increased expression of CD86 (*p* = 0.03), the CD11c^+^/CD86^+^ population doubled after maturation (*p* = 0.0003) (Figure 2C). A similar tendency was observed with the CD40 marker-maturation, resulting in the increased proportion of the CD11c^+^/CD40^+^ population (*p* = 0.005) and upregulation of CD40 expression (*p* = 0.003). Although maturation did not affect the size of the CD11c^+^/CD80^+^ population (*p* > 0.05), it induced a notable increase in the amount of surface CD80 molecules (*p* = 0.002). Prior to maturation, the majority of cells were CD11c^+^/PD-L1^+^, with a significant upregulation of PD-L1 on the surface of this population (*p* = 0.01). An increase in the proportion of the CD11c^+^/MHCII^high^ population, characterized by high levels of MHCII (*p* = 0.00003), was also observed. Further experiments were conducted using mature DCVs.

### 3.2. Survival Rates

**Figure 3:**
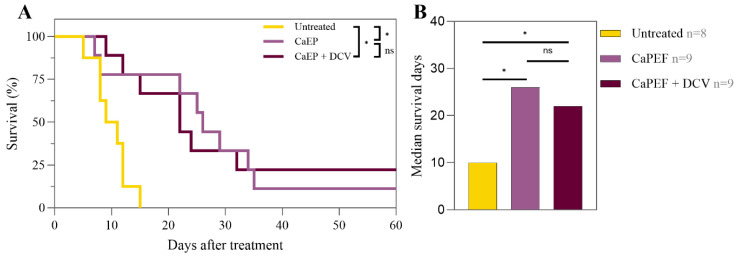
Kaplan–Meier survival curves (**A**) and median survival days (**B**) of mice with Lewis lung carcinoma tumors treated with CaPEF or CaPEF + DCV. Untreated—not treated tumor-bearing mice; EP protocol—1.3 kV/cm × 100 µs × 8 (1 Hz). The asterisk (*) highlights statistically significant differences (Mantel–Cox test; * *p* < 0.05), while “ns” is insignificant.

Furthermore, we assessed the treatment efficacy of DCV and CaPEF in the LLC1 tumor model by comparing survival rates (Figure 3).

A statistically significant increase in survival was noted in both treatment groups (CaPEF and CaPEF + DCV) in contrast to the untreated mice with tumors. The median survival time of untreated tumor-bearing mice was 10 days, while PEF-treated mice exhibited a more than two-fold increase in median survival time (CaPEF: 26 days; CaPEF + DCV: 22 days). The treatment of lung carcinoma with the CaPEF and DCV combination resulted in no significant changes in mice survival when compared to the CaPEF treatment group. Once the study had concluded, both treatment groups had fully recovered mice: CaPEF—1 mouse and CaPEF + DCV—2 mice.

### 3.3. Lymphocyte Subsets in Spleen and Lymph Nodes

**Figure 4:**
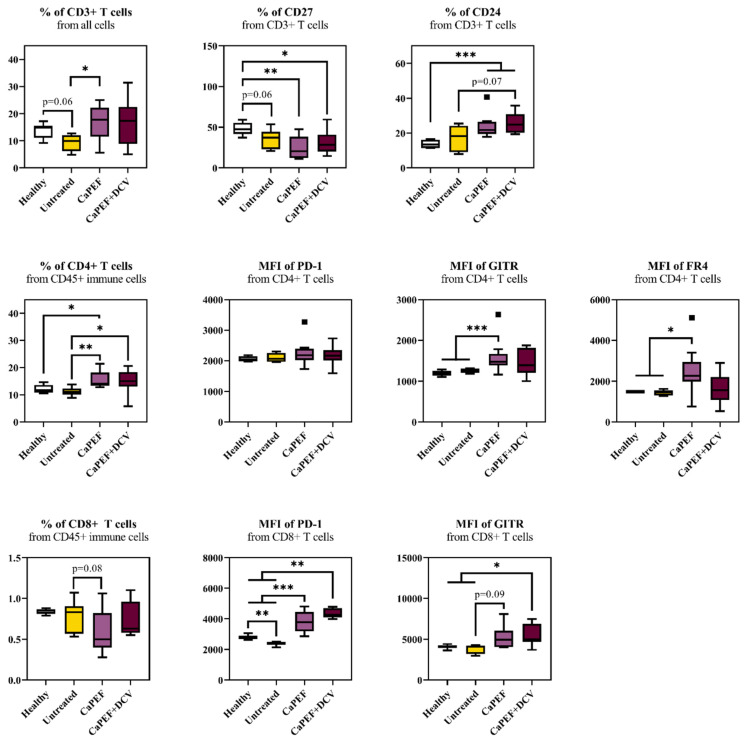
Analysis of splenic T lymphocyte subpopulations in Lewis lung carcinoma-bearing mice treated with CaPEF or CaPEF + DCV. Untreated—group consisting of tumor-bearing mice that did not receive any therapy. EP protocol—1.3 kV/cm × 100 µs × 8 (1 Hz). Flow cytometric analysis was carried out with a BD FACSAria III cytometer. The statistical comparison of lymphocyte subsets between experimental groups was conducted using the non-parametric Mann–Whitney U test. Statistically significant differences are indicated by black asterisks (*), with thresholds set at * *p* < 0.05, ** *p* < 0.005, *** *p* < 0.0005. Tendencies (*p* = 0.05–0.1) were evaluated as well. Black squares represent outliers in the Tukey boxplot.

Subsequent to the in vivo experiment, a comprehensive analysis of changes in immune cell subpopulations was performed. For this analysis, both spleen and lymph nodes were used from healthy, untreated (tumor-bearing), CaPEF and CaPEF + DCV treated C57BL/6 mice. Firstly, splenic T lymphocyte subpopulation changes were analyzed (Figure 4).

A statistically significant elevation in the proportion of CD3^+^ lymphocytes was observed in the CaPEF group compared to the untreated control (Figure 4). However, no significant differences were observed between the CaPEF and CaPEF + DCV treatments. At the same time, from the CD3^+^ cells, a relative percentage of Tumor Necrosis Factor-Receptor (TNF-R), CD27 was lower after PEF-based treatments when compared to the healthy animals. The opposite tendency was detected in the CD3^+^CD24^+^ cells subset—i.e., both CaPEF and CaPEF + DCV groups had a significantly higher percentage of CD24^+^ when compared to the healthy group.

Furthermore, CD4^+^ T cells and their surface marker changes were analyzed. From CD45^+^ immune cell subpopulation, a significantly higher increase in CD4^+^ in both CaPEF and CaPEF + DCV groups was observed when compared to the untreated group. The differences in the mean fluorescence intensity (MFI) of PD-1 in the CD45^+^CD4^+^ cell subpopulation were not statistically significant, while significantly higher MFIs of GITR and FR4 were detected. This was not the case for the CaPEF + DCV group—i.e., MFIs of PD-1, GITR, and FR4 in the CD45^+^CD4^+^ subpopulation were comparable with the healthy and untreated mice groups (*p* > 0.05).

Likewise, CD8^+^ subsets were examined, revealing no differences in the percentage of CD8^+^ cells within the CD45^+^ cell population. However, the increase in the MFI of PD-1 from CD45^+^CD8^+^ was significantly higher in both treatment groups in contrast to healthy or untreated mice. Simultaneously, the untreated group exhibited a significantly lower MFI of PD-1 from CD45^+^CD8^+^ than the healthy group. The MFI of GITR from CD45^+^CD8^+^ was elevated in both treatment groups; however, statistical significance was observed only in the CaPEF + DCV group when compared to either healthy controls or untreated tumor-bearing mice.

**Figure 5:**
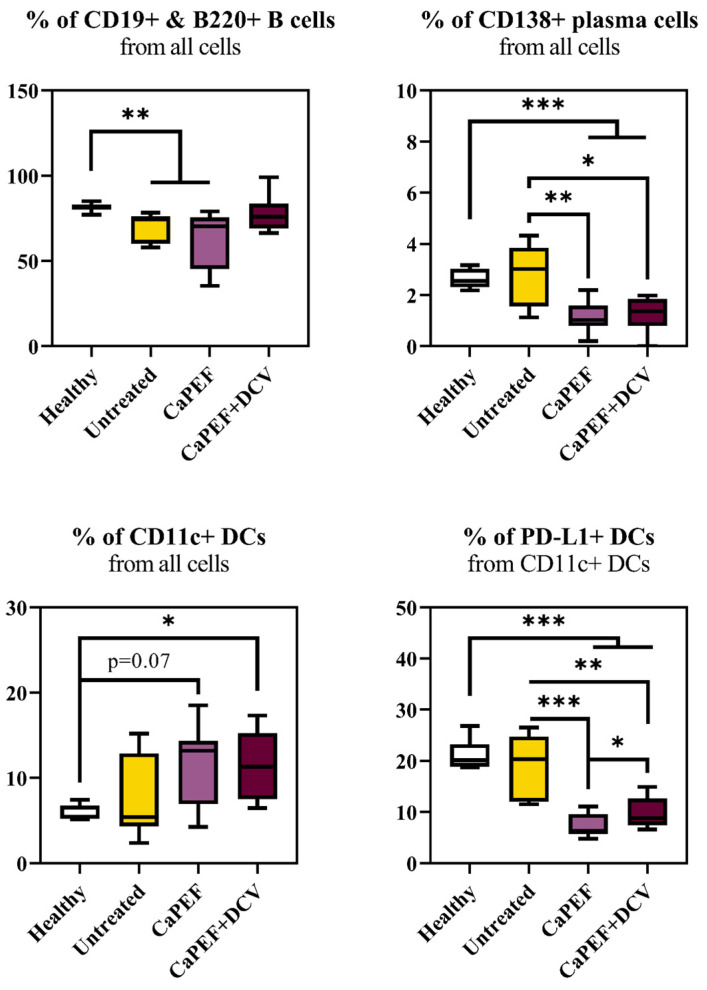
Splenic myeloid and B cell subpopulations of mice with Lewis lung carcinoma tumors treated with CaPEF or CaPEF + DCV. Untreated—group consisting of tumor-bearing mice that did not receive any therapy; EP protocol—1.3 kV/cm × 100 µs × 8 (1 Hz). Flow cytometric analysis was carried out with a BD FACSAria III cytometer. The statistical comparison of lymphocyte subsets between experimental groups was conducted using the non-parametric Mann–Whitney U test. Statistically significant differences are indicated by black asterisks (*), with thresholds set at * *p* < 0.05, ** *p* < 0.005, *** *p* < 0.0005. Tendencies (*p* = 0.05–0.1) were evaluated as well.

Further, the myeloid and B cells in the spleens were analyzed (Figure 5).

From splenic B cells, a significant percentage decrease in CD19^+^B220^+^ cells were acquired in both untreated and CaPEF groups when compared to the healthy group. Subsequently, there were no statistically meaningful changes in the CaPEF + DCV group, while a significant percentage decrease in CD138^+^ plasma cells in both PEF treatment groups was detected. Both PEF treatments had a tendency to increase the percentage of CD11c^+^ dendritic cells; however, the percentage of dendritic cells CD11c^+^PD-L1^+^ was significantly lessened in CaPEF and CaPEF + DCV groups when compared with the healthy or untreated mice groups.

**Figure 6:**
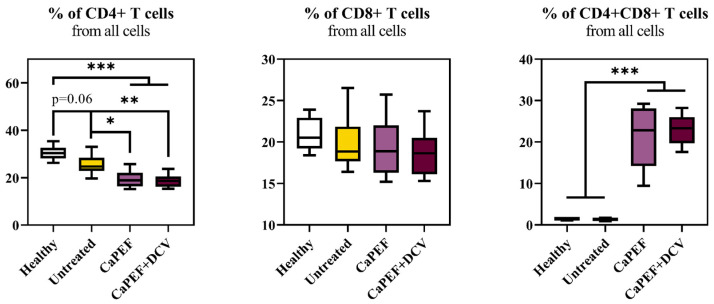
T lymphocyte subpopulations from lymph nodes of mice with Lewis lung carcinoma tumors treated with CaPEF or CaPEF + DCV. Untreated—not treated tumor-bearing mice; EP protocol—1.3 kV/cm × 100 µs × 8 (1 Hz). Cytometry was performed with a BD FACSAria III cytometer. The Mann–Whitney test was used to compare lymphocyte subset data. The black asterisk (*) denotes statistically significant (* *p* < 0.05, ** *p* < 0.005, *** *p* < 0.0005) differences between groups. Tendencies (*p* = 0.05–0.1) were evaluated as well.

Further, the T lymphocyte subpopulation changes in lymph nodes were analyzed (Figure 6).

Both CaPEF treatments resulted in significant percentage decrease in the CD4^+^ T cell subpopulation, but no differences in the percentage of CD8^+^ T cells subset when compared to the healthy or untreated mice groups. Additionally, a highly significant increase in the double positive T cells subset (CD4^+^CD8^+^) was detected when treatment involved CaPEF or CaPEF + DCV.

**Figure 7:**
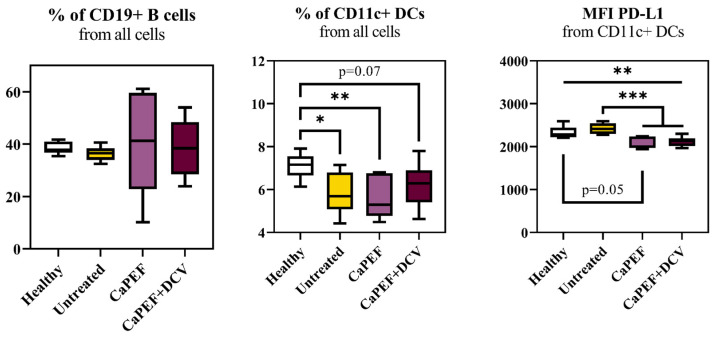
Myeloid and B cells subpopulations from lymph nodes of mice with Lewis lung carcinoma tumors treated with CaPEF or CaPEF + DCV. Untreated—not treated tumor-bearing mice; EP protocol—1.3 kV/cm × 100 µs × 8 (1 Hz). Cytometry was performed with a BD FACSAria III cytometer. The Mann–Whitney test was used to compare lymphocyte subset data. The black asterisk (*) denotes statistically significant (* *p* < 0.05, ** *p* < 0.005, *** *p* < 0.0005) differences between groups. Tendencies (*p* = 0.05–0.1) were evaluated as well.

Finally, changes in immune cell subpopulation were assessed by evaluating myeloid and B cells in mice lymph nodes (Figure 7).

No significant differences were observed in the percentage of CD19^+^ B cells across all groups; however, the percentage of CD11c^+^ DCs decreased in all groups when compared to healthy animals. The MFI of PD-L1 from CD11c^+^ DCs was lower in both groups involving PEF treatment when compared to the untreated mice. The decrease in the MFI of PD-L1 from CD11c^+^ DCs was also significantly lower when compared to healthy animals in the case of CaPEF + DCV treatment, but a trend (*p* = 0.05) was detected in the case of CaPEF only.

### 3.4. Antitumor Immune Response

**Figure 8:**
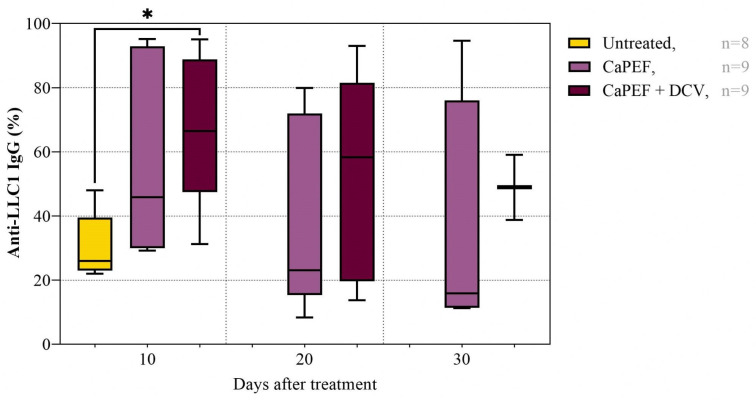
The relative percentage of anti-LLC1 IgG antibodies in mice sera, where Lewis lung carcinoma (LLC1) tumors were treated with CaPEF (n = 9) or CaPEF with DCV (CaPEF + DCV, n = 9). Untreated (n = 8)—not treated tumor-bearing mice; EP protocol—1.3 kV/cm × 100 µs × 8 (1 Hz). Negative control is at 0%. Blood was taken on the 10th, 20th, and 30th days after treatment. The Mann–Whitney test was used to compare antitumor IgG antibody data. The black asterisk (*) denotes statistically significant (* *p* < 0.05) differences between groups.

The relative percentage of anti-LLC1 IgG antibodies in C57BL/6 mice sera was determined (Figure 8).

The combination of both CaPEF and the mature DC vaccine (CaPEF + DCV), significantly increased antitumor antibody percentage when compared to the untreated mice group on the 10th day after treatment. There were no statistically significant differences between the CaPEF and CaPEF + DCV groups. A slight decrease in antitumor IgG antibody levels was observed on the 20th and 30th days after treatment, in comparison to 10 days. Additionally, it is important to note that fewer mice were present on days 20 and 30 due to survival rates (see Figure 3).

### 3.5. Survival Rates of the Second Experiment

**Figure 9:**
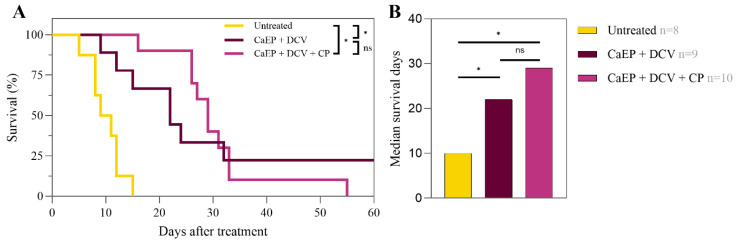
Kaplan–Meier survival curves (**A**) and median survival days (**B**) of mice with Lewis lung carcinoma tumors treated with CaPEF, CaPEF + DCV, and CaPEF + DCV + CP. Untreated—not treated tumor-bearing mice; EP protocol—1.3 kV/cm × 100 µs × 8 (1 Hz). The black asterisk (*) illustrates statistically significant differences using Log-rank (Mantel–Cox test; *p* < 0.05), while n.s. is not significant.

Taking into account the limited effect of the DCV in the first experiment, an additional pilot study using the LLC1 tumor model was conducted with the addition of the chemotherapeutic drug cyclophosphamide (CP), which has been reported to have an immunomodulatory role (Figure 1, Exp. 2). The results can be seen in Figure 9.

Compared to untreated tumor-bearing mice, the survival of both CaPEF + DCV and CaPEF + DCV + CP treated mice were significantly longer. The median survival time of untreated tumor-bearing mice was 10 days, whereas that of mice treated with CaPEF + DCV was 22 days. The addition of the CP to the CaPEF + DCV treatment extended median survival time to 29 days, but the differences were not statistically significant. Taking into consideration that using CP does not trigger statistically significant changes in survival rates of mice, no additional in vitro immunology experiments were performed in accordance with the available bioethics agreement.

## 4. Discussion

In this study, we evaluated the effects of microsecond-range calcium electrochemotherapy, either alone or in combination with a dendritic cell vaccine and/or cyclophosphamide. The primary objective was to assess whether the combination of CaPEF treatment with DCV could elicit additional immunomodulatory effects and trigger synergistic effects by improving overall therapeutic efficacy. Our findings demonstrate that microsecond-range calcium electroporation significantly prolonged survival rates, with no evidence of a positive synergistic interaction when combined with DCV. Notably, a systemic immune response was induced following both CaPEF and CaPEF + DCV treatments. Mice treated with CaPEF—either alone or with DCV—exhibited improved survival when compared to untreated tumor-bearing mice. However, the combination with DCV did not result in significant improvements. The prepared DCVs were characterized by high purity and the expression of molecules necessary for T lymphocyte stimulation. One of the reasons for the lack of a positive effect could be the high expression of the immunoregulatory molecule PD-L1 on the surface of DCs. By binding to the PD-1 molecule on T lymphocytes, PD-L1 inhibits their proliferation, activation, and cytotoxic properties. This protects against autoimmune reactions in healthy conditions; however, in cancer, the PD-L1/PD-1 interaction protects tumor cells from destruction by CD8 T lymphocytes, thereby supporting their growth [80]. The PD-L1 is broadly expressed across immune cells, including immune cells such as T and B lymphocytes, NK cells, macrophages, and dendritic cells [81]. Although PD-L1 expression is typically low in these cells, inflammatory factors, such as IFNg, IFNa, IFNb, LPS, IL-4, and GM-CSF [82] as well as immunosuppressive factors [83] from tumors, induce PD-L1 expression. Their stimulatory effects are the basis of many protocols to produce murine DCs from bone marrow [84], only their composition and amounts vary between studies. Some studies based on analogous protocols also observed high PD-L1 and/or PD-L2 expression on the surface of prepared DCs. Nevertheless, such DCs were able to stimulate potent T lymphocyte responses both in vitro [85] and in vivo, also demonstrating antimetastatic potential [86]. PD-L1 expression on DCs is essential for protecting them from the cytotoxic T lymphocytes but may also negatively affect the antitumor response [87]. Studies show that combining DCVs with Abs targeting the PD-L1/PD-1 axis can significantly increase its effectiveness. For example, pretreatment of mouse splenic DCs with anti-PD-L1 mAbs led to improved T lymphocyte proliferation and effector properties. In the same study, renal cell or non-small cell lung carcinoma patients, whose tumors had high expression of DC-associated genes, responded better to treatment with atezolizumab (anti-PD-L1), where the treatment significantly improved overall survival compared to patients whose tumors had low expression of these genes [88]. In another study, deletion of PD-L1 in DCs led to slowed tumor growth and improved antitumor CD8 T lymphocyte responses in a mouse tumor model [89]. To summarize, the contributing factors for the lack of DCV effects in our study may include the DCV formulation protocol [90], antigen selection for DCV maturation [91], the necessity of combination therapies [92], and the role of PD-L1 blockade in established tumors to enhance T cell activation [87]. Other studies have also reported limited efficiency of DCV treatment, which results in inconsistent clinical responses in patients [93]. It has been reported that DCV-based treatment modalities can yield low response rates, i.e., only 9.3% of lung cancer patients have shown objective responses [91].

Therefore, after the unsatisfactory efficiency of CaPEF + DCV in our study, we combined the treatment with CP as an effort to potentiate the therapeutic outcome of CaPEF + DCV (Figure 9). While such a strategy improved the survival rate of animals (29 days versus 22), the differences were still not statistically significant. Lack of synergistic effect may be attributed to low immunogenicity of 4T1 cell line and the big volume of the tumor at the time of treatment (Day 0). Large tumor volumes are known to impair the effectiveness of DCVs. Lack of a synergistic effect could also be influenced by the high expression of the immunoregulatory molecule PD-L1 on the surface of the DCs. By binding to the PD-1 molecule on T lymphocytes, PD-L1 inhibits their proliferation, activation, and cytotoxic properties. This protects against autoimmune reactions in healthy conditions; however, in cancer, the PD-L1/PD-1 interaction protects tumor cells from destruction by CD8 T lymphocytes, thereby supporting their growth [80]. Finally, our study employed intraperitoneal administration of the DCV to promote systemic immune activation, as supported by prior studies [94]. Other administration routes such as subcutaneous (SC) [94], intradermal (ID) [95] or intratumoral (IT) [94] can be investigated in the future in combination with CaPEF. In our study, the intraperitoneal route was selected for its proximity to the tumor site and to avoid the post-PEF scab on the primary tumor site, but it is likely that treatment outcome reflects a combination of tumor immunogenicity, tumor burden, treatment timing, and route of administration.

Nevertheless, both PEF-based therapies in our study induced significant changes in immune cell populations. In untreated tumor-bearing mice, splenic CD3^+^ T cells were significantly reduced compared to healthy controls, while the effect was reversed by both CaPEF and CaPEF + DCV treatments. CD27 expression—a costimulatory molecule vital for T cell memory [96,97,98], was reduced in all tumor-bearing groups, including treated ones. Increased PD-1 expression on CD8^+^ T cells may reflect TCR/CD3 pathway inhibition due to PD-1-mediated suppression.

A modest increase in CD24 expression was observed in the CD3^+^ T cell population following CaPEF + DCV treatment. CD24 is associated with homeostatic T cell proliferation and antigen-driven expansion in both lymphoid and non-lymphoid organs [99,100]. Additionally, CaPEF, with or without the DCV, elevated the expression of GITR on CD8^+^ T cells, which may enhance antitumor immunity by suppressing regulatory T cells and promoting helper T cell (Th9) differentiation [101]. T cell exhaustion was further indicated by increased frequencies of CD4^+^CD8^+^ double-positive T cells in lymph nodes. Notably, CD4^+^ T cell levels rose in the spleen and declined in lymph nodes, likely due to prolonged antigen exposure and T cell redistribution, potentially leading to splenomegaly [102]. The observed upregulation of folate receptor 4 (FR4) on regulatory T cells could result from T cell exhaustion or the immunosuppressive nature of recurrent tumors [103,104,105]. Additionally, an increase in dendritic cells (CD11c^+^) was observed in the spleens, accompanied by a reduction in the lymph nodes of treated mice. PD-L1 expression was reduced in both spleen and lymph nodes following PEF treatments; however, splenic DCs from the CaPEF + DCV group exhibited significantly higher PD-L1 levels than those from the CaPEF group.

The humoral antitumor response was also affected by PEF treatment, i.e., 10 days after treatment antibody levels were elevated in both CaPEF and CaPEF + DCV groups, suggesting a boosted humoral antitumor response. This effect was particularly pronounced in the CaPEF + DCV group, presumably due to enhanced CD4^+^ T cell priming, leading to B cell activation and plasma cell differentiation [106].

## 5. Conclusions

Our results demonstrated that DCV injection in combination with microsecond range calcium electrochemotherapy did not induce statistically significant synergistic effects. We underline several possible limitations: (1) a low-immunogenicity tumor model employed; (2) intraperitoneal DCV injection was used (due to close proximity to the tumor and the post-PEF scab) while other injection sites could have been selected; (3) if PD-L1 expression on the DC surface is the reason for lack of synergy, there are two possible ways to improve treatment efficacy—either further optimize the DCV preparation protocol and/or include mAbs that inhibit PD-L1/PD-1 signaling.

## Data Availability

Data available from the corresponding author E.R.-V. on request.

## Supplementary Materials

The following supporting information can be downloaded at: https://www.mdpi.com/article/10.3390/pharmaceutics17070804/s1, Supplementary Table S1: List of antibodies (Ab) used; Supplementary Figure S1: Gating strategy for flow cytometry data.
