# Supplementary material for: Calcium Electrochemotherapy and Challenges in Combined Treatment with Dendritic Cell Vaccination
Source: Pharmaceutics. 2025 Jun 21;17(7):804. doi: 10.3390/pharmaceutics17070804 (PMC12299573; doi:10.3390/pharmaceutics17070804)
Supplement: Supplementary file 1 [file pharmaceutics-17-00804-s001.zip › pharmaceutics-3689086-supplementary.pdf]

Supplementary Material

Calcium Electrochemotherapy and Challenges in Combined Treatment with Dendritic Cell Vaccination

Supplementary Table S1. List of antibodies (Ab) used.

Conjugated Fluorochromes	Anti-Mouse Ab Against Cell Markers	Manufacturer	Catalog Identifier #
AF488	IgG	Invitrogen	2379467
AF700	CD8	Invitrogen	2075802
APC	CD138	Invitrogen	800-874-3723
	CD11b	Miltenyi Biotec	120-001-709
	CD25	Invitrogen	12-0251-81
APC-Cy7	CD8	Molecular probes	A15386
BV421	FR4	BD	744119
eFluor™ 450	CD40	Invitrogen	48-0402-82
	CD44	Invitrogen	48-0441-82
	Ly-6C	Invitrogen	48-5932-82
eFluor™ 506	CD19	eBioscience™	69-0193-82
	PD-1	Invitrogen	69-9985-82
FITC	Gr1	Miltenyi Biotec	120-002-248
	CD3	Invitrogen	11-0038-42
PE	Gr1	Biolegend	108407
	CD31	BD	553373
	B220	BD	553090
PE-TxRed	CD11b	Invitrogen	RM2817
	CD11c	Invitrogen	MCD11C17
PerCP	CD45	BD	2329575
PerCP-Vio700	CD4	Miltenyi Biotec	130-102-271

Conjugated Fluorochromes	Anti-Mouse Ab Against Cell Markers	Manufacturer	Catalog Identifier #
PerCP-eFluor™ 710	CD24	Invitrogen	2169304
SuperBright 600™	CD27	Invitrogen	2212412
	CD357	Invitrogen	63-5874-82

Anti-mouse FcγR (Fc block) – supernatant from hybridoma cells secreting monoclonal antibodies against Fc receptor (home-made, CIM, Vilnius, Lithuania).

LIVE/DEAD™ Fixable Near-IR Stain #L10119 (Thermo Fisher Scientific, USA).

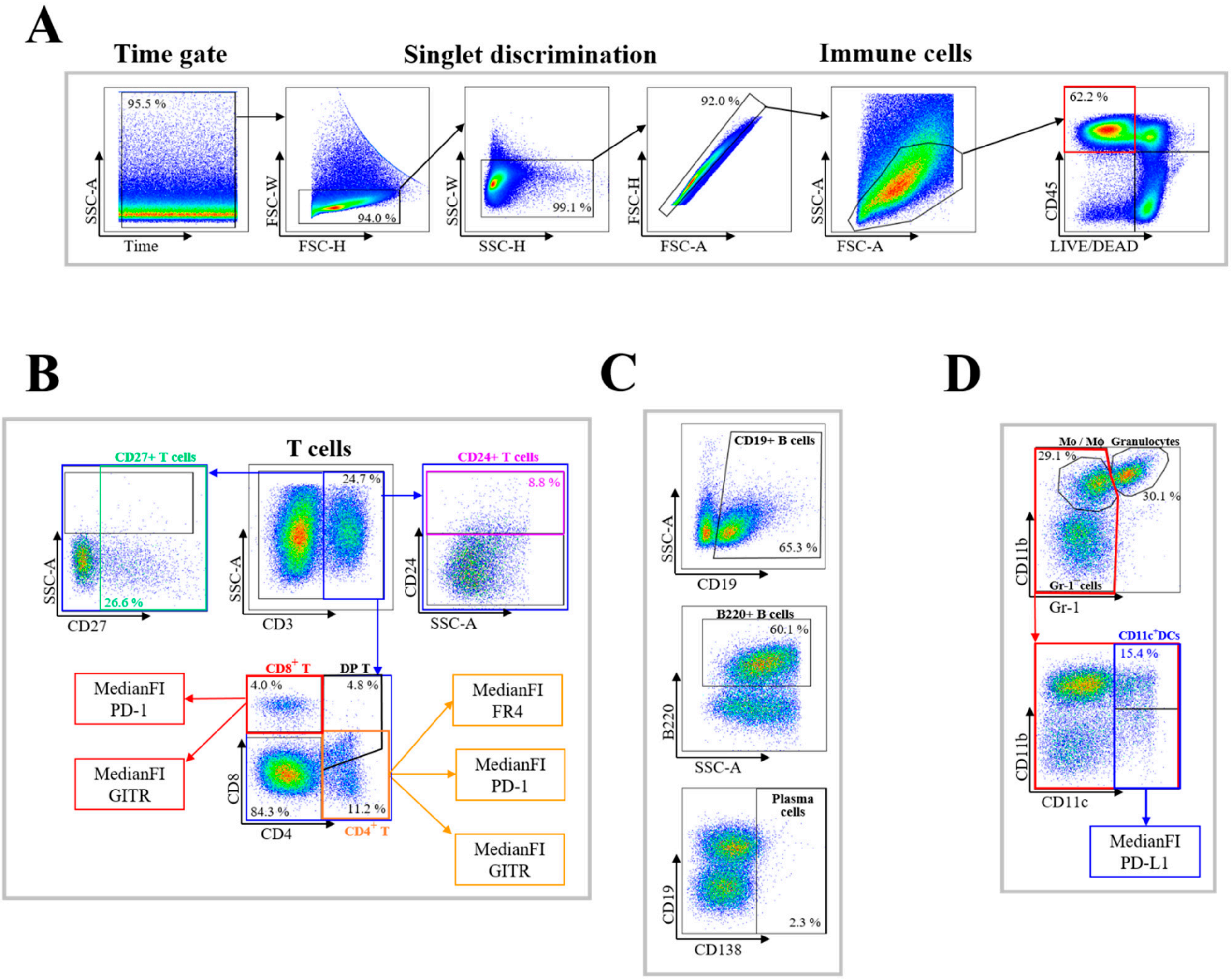

Figure S1. Gating strategy for flow cytometry data. **A**) A time gate was initially applied to eliminate events influenced by aberrations during sample acquisition. Doublets were discriminated, and live and immune cells were further gated; **B**) Lymphocytes: $CD8^+$ T, $CD4^+$ T cells and the median fluorescence intensity (MedianFI) of surface markers; **C**) Lymphocytes: B and plasma cells; **D**) Myeloid cells: dendritic cells and MedianFI of PD-L1.
